# Epigenetic Modifications and Diabetic Retinopathy

**DOI:** 10.1155/2013/635284

**Published:** 2013-10-28

**Authors:** Renu A. Kowluru, Julia M. Santos, Manish Mishra

**Affiliations:** Kresge Eye Institute, Wayne State University, 4717 St. Antoine, Detroit, MI 48201, USA

## Abstract

Diabetic retinopathy remains one of the most debilitating chronic complications, but despite extensive research in the field, the exact mechanism(s) responsible for how retina is damaged in diabetes remains ambiguous. Many metabolic pathways have been implicated in its development, and genes associated with these pathways are altered. Diabetic environment also facilitates epigenetics modifications, which can alter the gene expression without permanent changes in DNA sequence. The role of epigenetics in diabetic retinopathy is now an emerging area, and recent work has shown that genes encoding mitochondrial superoxide dismutase (*Sod2*) and matrix metalloproteinase-9 (*MMP-9*) are epigenetically modified, activates of epigenetic modification enzymes, histone lysine demethylase 1 (LSD1), and DNA methyltransferase are increased, and the micro RNAs responsible for regulating nuclear transcriptional factor and VEGF are upregulated. With the growing evidence of epigenetic modifications in diabetic retinopathy, better understanding of these modifications has potential to identify novel targets to inhibit this devastating disease. Fortunately, the inhibitors and mimics targeted towards histone modification, DNA methylation, and miRNAs are now being tried for cancer and other chronic diseases, and better understanding of the role of epigenetics in diabetic retinopathy will open the door for their possible use in combating this blinding disease.

## 1. Introduction

Diabetic retinopathy remains the leading cause of blindness in young adults affecting over 90% patients with 20 years of diabetes. The disease carries a heavy burden on our society as it is responsible for 4.8% of the 37 million cases of eye disease-related blindness worldwide. With the incidence of diabetes increasing at an alarming rate, the number of people with diabetic retinopathy is expected to grow from 126.6 million in 2010 to 191.0 million by 2030 [1]. This slow progressing disease is mainly characterized by damage of the microvasculature of the retina. Some of the early histopathological changes of this slow progressing disease include microaneurysms, hemorrhages, cotton wool spots, intraretinal microvascular abnormalities, and venous bleeding. But, in more advanced stages, new fragile vessels are formed along the retina and on the posterior surface of the vitreous, and if not treated, they result in the detachment of the retina, leading to blindness [2]. Although hyperglycemia is the major initiator of diabetic retinopathy, hypertension and dyslipidemia are also considered significant risk factors in its development and progression [3–5].

 Despite extensive research in the field to understand the pathogenesis of diabetic retinopathy, the exact mechanism(s) remains elusive making inhibition of the progression of this disease a daunting task. Some of the possible mechanisms implicated in its development include oxidative stress, increased formation of advanced glycation end products, and activation of protein kinase-C, polyol production, and hexosamine pathways [2, 6, 7]. These pathways appear to be interlinked [8], which further complicates the strategies to prevent the development/progression of this devastating complication of diabetes.

## 2. Genetics and Diabetic Retinopathy

Pathogenesis of a disease is also influenced by genetic factors, and due to variability in the severity of retinopathy among diabetic patients with similar risk factors, the role of genetic factors in diabetic retinopathy should be somewhat predictable. However, such genetic associations are not yet clearly established. Landmark “Genome-Wide Association Studies” have identified number of genetic variants that could explain some of the interindividual variations in the susceptibility of diabetes [9]. A meta-analysis study to investigate possible genetic associations with diabetic retinopathy, which incorporated reports published between January 1990 and August 2008, has revealed a significant variation in the *AKR1B1* gene. This gene encodes aldo-keto reductase family 1 member B1, which is the rate limiting enzyme of the polyol pathway [10], one of the pathways implicated in the development of diabetic retinopathy [2, 6, 7]. Another meta-analysis study has shown that Ala allele of the Pro12Ala polymorphism in the peroxisome proliferator-activated receptor-*γ*2 gene acts as a protective gene in the incidence of retinopathy in type 2 diabetic patients [11]. In contrast, recent studies have failed to find any associations between VEGF-related SNPs (rs6921438 and rs10738760) and the risk of diabetic retinopathy and nephropathy in diabetic patients [12]. Thus, the association between genetic factors and diabetic retinopathy remains to be clarified.

## 3. Epigenetic Modifications and Gene Regulation

Diabetic environment disturbs metabolic homeostasis and also alters various genes, including genes associated with oxidative stress, apoptosis and inflammation [13–16]. Control of gene expression (i.e., the ability of a gene to produce a biologically active protein) in mammals, in addition to being modulated by transcriptional and translational initiation, can also be controlled by changes “on the top” of the genes without altering the nucleotide composition of the genome [17]. These “epigenetic” modifications are stable, but potentially reversible, and can be passed from generation to generation. Recent studies have shown that epigenetic changes play a major role in many chronic diseases such as cancer and diabetes where small changes in the epigenome over time are considered to lead to disease manifestation. Three major epigenetic mechanisms considered to regulate gene expression are DNA methylation, histone modifications, and noncoding RNA activity [18–21].

 DNA methylation, addition of a methyl group on position 5 of cytosine residues of the cluster of CpG dinucleotides (CpG Island), which is the regulatory region of most genes, is typically associated with transcriptional repression [22, 23]. The methylation process brings in the unintended changes brought up by the environmental exposures or other life style, and these changes can be passed on for multiple generations. Cytosine 5 methylation generates 5-methylcytosine (5mC), and the reaction is catalyzed by a family of enzymes—DNA (cytosine-5) methyltransferases (Dnmts). The conversion of 5mC to 5-hydroxymethylcytosine (5hmC) is facilitated by Ten-eleven-translocation enzymes (TETs) [24, 25], making these enzymes future targets of pharmacological regulation. However, how the process of DNA methylation-demethylation is balanced remains somewhat unclear. A subfamily of DNA glycosylases are considered to promote active DNA demethylation by removing the 5-methylcytosine base, followed by cleavage of the DNA backbone at the abasic site, and the methylated cytosine is replaced by an unmethylated cytosine. In contrast, the passive process involves absence/inactivation of Dnmt1 resulting in hypomethylated DNA [26].

 Gene expression pattern is also dictated by chromatin, which is a composite structure of histones and nucleic acid. Histones act as spools around which DNA winds, and among the 4-histone proteins, histone 2A and B (H2A and H2B), H3 and H4 form a tetrameric structure, the nucleosome [27]. Despite such sophisticated DNA packaging, N-terminal of histones remains vulnerable for posttranslational modifications, and can be acetylated, methylated, and phosphorylated. Such epigenetic modifications alter the chromatin structure which subsequently affects the binding of transcription factors, and can regulate the selective expression of genes in a particular tissue by acting like switches to control gene activity [15, 28–31].

 Acetylation is one of the most common modifications which is generally associated with gene activation, and the process relaxes the chromatin structure allowing recruitment and binding of transcription factor and RNA polymerase II [32]. Acetylation is regulated by fine balance between histone acetylating and deacetylating enzymes; histone acetyltransferases (HATs) add acetyl group while histone deacetylase (HDAC) removes the acetyl group. Methylation of histones, however, shows greater variability as methylation can occur at both lysine and arginine residues, and can be associated with either gene activation or repression. Methylation of lysine 4 of histone 3 (H3K4) is typically associated with gene activation, while methylation of lysine 9 (H3K9) is associated with gene repression [33–35]. Furthermore, the outcome of methylation is also dictated by the specific histone residue being modified; for example, mono- or tri-methylation of H3K4 induces activation of gene expression [36, 37]. While monomethylation of H3K9 induces gene activation, its tri-methylation suppresses the gene activity [38]. As with acetylation, methylation status of histones depends on the final balance between histone methylating and demethylating enzymes. Histone methylating enzymes, SET1/7/9 help in H3K4 methylation, and suppressors of variegation 3-9 homolog (SUV39H) methylates lysine 9 of histone 3 [39, 40]. Subsequently, in order to protect from such kind of relatively stable methyl modifications and constitutive expression of genes, lysine specific demethylase (LSD1) specifically removes methyl groups from methylated H3K4 and H3K9 [41, 42]. 

 In addition to DNA methylation and histone modifications, gene expression is also regulated by microRNAs (miRNA); the small noncoding RNAs that regulate gene expression posttranscriptionally by binding to complementary sequences in the 3′ untranslated regions of messenger RNAs [43, 44]. This cleaves mRNA resulting in decreased protein synthesis and expression of the targeted gene. Genes with epigenetic functions in chromatin can be regulated by miRNAs affecting chromatin remodeling and gene expression. Regulation of genes by DNA methylation and miRNA appears to be interrelated as the function of Dnmts depends on histone modification patterns, such as H3K9 methylation and histone deacetylation. In addition, inhibition of Dnmts, which causes DNA demethylation, can reactivate some of the miRNAs [45]. Overall, these epigenetic modifications are not permanent, but their continuous response to the changing environment, such as diabetes, and the chances of them being inherited to successive generation, make them attractive targets for chronic diseases.

## 4. Role of Epigenetic Modifications in Diabetes

It has become clear that both genetic predisposition and environmental factors play critical roles in the development of metabolic diseases including obesity and type 2 diabetes, and epigenetic modifications have important roles in altering gene expressions in various chronic diseases [46–48]. Recent studies have demonstrated the role of epigenetic mechanisms in islet function, and have provided an avenue whereby dietary components could accelerate or prevent age-related diseases through their effects on epigenetic modifications [49, 50]. Increased DNA methylation at the promoter of the peroxisome proliferator-activated receptor coactivator 1 gene in the pancreatic islets is shown to play a key role in regulating mitochondrial genes [51]. Our studies have shown that in diabetes hyper methylation of the CpG sites at the regulatory region of DNA polymerase gamma affects its binding to the mtDNA, and this compromises the transcriptional activity resulting in decreased copy number [52]. Hyper acetylation of histone H4 at the insulin gene promoter influences glucose-induced insulin secretion [53]. Diabetic patients with nephropathy have altered DNA methylation at the key gene promoters compared to those without nephropathy [54]. Tissue specific DNA methylation is observed in streptozotocin-induced diabetic rats with hypomethylation in the liver but not in kidney, and in the leukocytes of diabetic patients, Dnmt is altered [55, 56]. Furthermore, hyperglycemia induces specific and long-lasting epigenetic modifications [14, 15, 28, 29, 52, 57, 58]; glucose-induced persistent transcriptional activation of p65 in vascular cells is associated with methylation of H3K4 and hypomethylation of H3K9, and the concomitant activation of NF-*k*B is subsequently associated with increased inflammatory response [14].

 Studies using zebrafish as a model of diabetes have shown that hyperglycemia induces global DNA hypo methylation and aberrant gene expression in the tissue after limb amputation, and the process is associated with poor wound healing process [59]. Furthermore, *β*-cells from young IUGR animals, a model of type 2 diabetes, present changes in DNA methylation resulting in dysregulation of genes associated with cellular memory of intrauterine that is associated with adult susceptibility to diabetes [60]. Thus, epigenetic changes can account for chronic and persistent complications of diabetes.

 Diabetes also affects miRNAs; it is shown to downregulate miR133a, the miRNA which is implicated in the regulation of the key genes associated with cardiomyocyte hypertrophy [61]. In contrast, upregulation of miR-320 is observed in cardiac microvascular endothelial cells in type 2 diabetic rats [62]. miR-25, the miRNA which targets NADPH oxidase 4, is implicated in the pathogenesis of diabetic nephropathy via promoting oxidative stress [63]. In addition, decreased levels of miR-192 are also linked with the severity of nephropathy and fibrosis in diabetic patients [64]. Better understanding of regulatory roles of miRNAs in diabetic cardiomyopathy and other disease processes is expected to elucidate new avenues for RNA-based therapeutics.

## 5. Diabetic Retinopathy and Epigenetic Modifications

Diabetic retinopathy is a multifactorial disease and a number of metabolic abnormalities have been associated with its development [7, 65]. However, the role of epigenetic modifications in diabetic retinopathy is still not clear. *SUV39H2* is a gene that encodes histone methyltransferase which catalyzes the methylation of H3K9, and recent results from the Finnish Diabetic Nephropathy Study with ~3000 diabetic patients have found an association between the polymorphism in *SUV39H2* and diabetic microvascular complications, including retinopathy. These studies have suggested the role of histone modifications in the development of diabetic retinopathy [66]. Experimental evidence using in vitro and in vivo models of diabetic retinopathy have shown that the activities of HDACs are increased and that of HATs are decreased in the retina and its capillary cells in diabetes, and global acetylation of histones is decreased [58]. However, contrary to this, Kadiyala et al. have shown significant increase in retinal histone acetylation in diabetes [67]; strengthening further investigation into the role of histone modifying enzymes in the development of diabetic retinopathy.

 Mitochondria are dysfunctional in the retina and its capillary cells in diabetes, and superoxide radicals are elevated and the enzyme responsible for scavenging superoxide radicals in the mitochondria, MnSOD, is impaired [68–70]. Regulation of mitochondrial superoxide levels by maintaining MnSOD protects capillary cell apoptosis and the development of diabetic retinopathy in mice [69, 71]. In the pathogenesis of diabetic retinopathy, *Sod2*, the MnSOD encoding gene, is epigenetically modified with increased H4K20me3, acetyl H3K9, and p65 subunit of NF-*k*B (p65) at its promoter/enhancer. We have shown that increased acetyl H3K9 and p65 at *Sod2* interact with H4K20me3, and this results in its inactivation [15]. However, diabetes demethylates H3K4, and the recruitment of LSD1 at *Sod2* promoter is increased [28]. These results have strongly suggested that the demethylation of H3K4 by LSD1 is also an important factor in the downregulation of *Sod2*. Furthermore, overexpression of MnSOD prevents increase in H4K20me3 at *Sod2* suggesting that superoxide radicals have regulatory role in the epigenetic modification of *Sod2* and SUV4-20h2 [15]. This raises the possibility to utilize therapeutic modalities targeted towards regulation of methylation status of histones to prevent inhibition of MnSOD and protect mitochondrial damage.

Matrix metalloproteinase-9 (MMP-9), an enzyme which is proapoptotic in the development of diabetic retinopathy, is also epigenetically modified in the retina in diabetes [29, 72, 73]. We have shown that at its promoter, H3K9me2 is decreased, and acetyl H3K9 and the recruitment of LSD1 and p65 of NF-*k*B are increased. In addition, regulation of LSD1 ameliorates decreases in H3K9me2 and increases in p65 at *MMP-9* promoter, prevents the activation of MMP-9, and also inhibits the cell apoptosis. The results have suggested that the activated LSD1 hypomethylated H3K9 at *MMP-9 *promoter and this frees up the lysine 9 for acetylation. Acetylated H3K9 opens up the chromatin, and the accessibility to recruit p65 is increased [29]. This ultimately culminates in the activation of MMP-9 and mitochondria damage. Thus, the regulation of LSD1 by molecular or pharmacological means could possibly have potential to prevent/retard the development and progression of retinopathy by regulating *Sod2 *and *MMP-9* and preventing mitochondrial damage and the accelerated capillary cell apoptosis in diabetic patients. Epigenetic modifications of thioredoxin interacting protein, an endogenous inhibitor of antioxidant thioredoxin, are considered responsible for sustained *Cox2* expression seen in the retina in diabetes [74]. Furthermore, in diabetes, the CpG sites at the regulatory region of DNA polymerase gamma are hypermethylated affecting their binding to the mtDNA, and the activity of Dnmt is increased [52]. Because of this modification, the transcriptional activity is compromised and copy number is decreased. Thus, there is growing evidence that histone modifications and DNA methylation play an important role in the development of diabetic retinopathy.

 Alterations in miRNA expression have also been observed in diabetic eyes, and miRNAs in the eye, including miR200b (one of the VEGF regulating miRNAs) is downregulated [75]. Diabetes also upregulates some of the key NF-*k*B responsive miRNAs including miR-146, miR-155, miR-132, and miR-21 [76]; upregulation of miR-29b in the early stages of diabetes is considered to be protective against apoptosis of the retinal ganglion cells [77]. These diabetes-induced alterations in miRNAs suggest that they can be used as biomarkers to detect early stages of the disease progression.

 Thus, understanding and characterizing the epigenetic regulators and their role in the pathogenesis of diabetic retinopathy could help identify novel targets to combat this disease which is the major cause of blindness in young adults.

## 6. Oxidative Stress and Epigenetic Modifications

**Figure 1:**
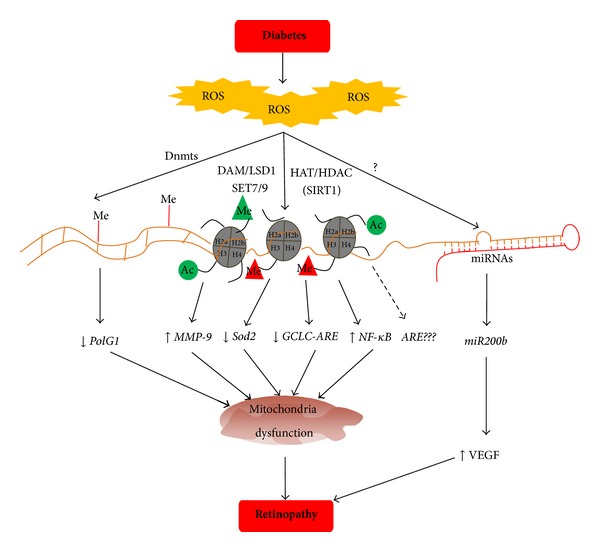
Epigenetic modifications and diabetic retinopathy: diabetes increases oxidative stress in the retina, reactive oxygen species (ROS) modify enzymes responsible for DNA methylation (Dnmts) and histone modifications (SAT, LSD1, HAT and HDAC, etc.), and also miRNAs. Due to epigenetic modifications of various genes and transcription factors, the expression of the targeted genes is altered resulting in mitochondrial dysfunction, and ultimately, in the development of diabetic retinopathy.

 The disruption of the normal redox potential in a cellular environment has detrimental effects on cellular components, including proteins, lipids, and DNA [78–80]. Oxidative stress has been shown to alter histone acetylation/deacetylation and methylation/demethylation. Histone demethylase LSD1 is FAD-dependent amine oxidases, and requires oxygen to function [81]. Oxidative damage to DNA can also result in epigenetic changes in chromatin organization by inhibiting the binding of the methyl-CpG binding domain of methyl-CpG binding protein 2 [82]. Furthermore, disruption of epigenetic mechanisms can result in oxidative stress [83]. In the pathogenesis of diabetic retinopathy, increased oxidative stress is considered to play a major role [7, 65], and our studies have shown that the control of oxidative stress prevents epigenetic changes in retinal *Sod2* [15]. Thus, it is plausible that increased oxidative stress could be one of the mechanisms responsible for modulating epigenetic modifications in diabetic retinopathy (Figure 1).

## 7. Epigenetic Modifications of Mitochondrial DNA

Although epigenetic changes in nuclear DNA (nDNA) are now emerging as important areas of investigation in many acquired chronic diseases, epigenetic modification of mtDNA is still in its incipient stages. Recent work has shown that the common epigenetic modifications in nDNA, such as the presence of 5mC and 5hmC, are also observed in the mtDNA, and mitochondria are also equipped with Dnmts and TETs [84]. Furthermore, mtDNA methylation decreases with age, and this age-related hypo methylation is linked with the decreased transcription capacity of proteins encoded by mtDNA [85]. Increase in Dnmt1 in the mitochondria is shown to upregulate the first mtDNA-encoded gene after its initiation, *ND1*, and downregulate *Cytochrome b,* the last gene encoded by mtDNA [84]. How diabetes affects mtDNA methylation and the transcription remains to be investigated.

In addition to dysfunction of the mitochondria and their structural abnormalities, mtDNA is also damaged in diabetes, and the DNA polymerase gamma (POLG1), a key member of the DNA replication machinery, is downregulated [13, 52, 71, 86, 87]. The CpG sites at the regulatory region of *POLG* in the retina are hypermethylated, and this possibly compromises its transcriptional activity [52].

Mitochondrial DNA is very small in size and does not contain histones; instead this DNA is covered with proteins, mainly the mitochondrial transcriptional factor A (TFAM), to form nucleoid [88]. In diabetic retinopathy, the binding of TFAM to a nonspecific region of mtDNA is decreased resulting in decreased levelsn of mtDNA-encoded proteins, and damaging the mtDNA [89, 90]. Since posttranslational modification of histones affects nDNA transcription, it is plausible that such changes in TFAM structure might affect mtDNA transcription. This suggests that by maintaining mitochondria homeostasis by modulating epigenetic changes using pharmaceutical or molecular means could help retard further progression of diabetic retinopathy.

## 8. Therapies Targeting Epigenetic Modifications

It is now becoming clear that epigenetic factors have a role in altering gene expression in chronic diseases, suggesting that epigenetic mechanisms play a pivotal role in an acquired phenotype. As mentioned above, DNA methylation is one of the most common epigenetic modifications, and methylation at specific CpG islands can provide insights into disease diagnosis. Thus, methylation of genes associated with that disease can be used as a predictor of efficacy for particular drug treatments. The most commonly used DNA methylation inhibitors are nucleoside analogs; these analogs incorporate into the DNA and trap all DNA methyltransferases. Fortunately, FDA has approved Dnmt inhibitors 5-azacytidine (5-Aza-CR; azacitidine; Vidaza) and 5-aza-20-deoxycytidine (5-Aza-CdR; decitabine; Dacogen) for myeloid cancers and cutaneous T cell lymphoma. Furthermore, DNA demethylation agent can also enhance the sensitivity of highly chemoresistant cells to chemotherapeutic drugs, suggesting their use as a promising approach in treating certain cancers [91]. However, these agents have significant toxicity at high concentrations due to DNA damage, and a group of more potent and selective Dnmt inhibitors, potentially with less toxicity, is now being under development. Zebularine (1-(*β*-D-ribofuranosyl)-2(1H)-pyrimidinone), a cytidine lacking 4-amino group of the pyrimidine ring, is less toxic and acts by forming a covalent complex with Dnmt and cytidine deaminase when incorporated into DNA [92]. In addition, recent work has shown that the status of methylation of VEGFR promoter dictates the efficacy of the VEGF-targeted drugs on the proliferation of cancer tissue, suggesting that the epigenetic alteration of VEGFRs could influence the efficacy of VEGF-specific tyrosine kinase inhibitors [93]. This is very significant for diabetic patients experiencing possibility of losing vision due to retinopathy as VEGF is considered as one of the major growth factor in the neovascularization associated with proliferative diabetic retinopathy.

 As mentioned above, histone modifications can alter gene expression modifying the risk for a disease, and the process is homeostatically balanced by groups of cellular enzymes that add an acetyl or methyl group or remove them. Epigallocatechin-3-gallate is a strong HAT inhibitor, and it inhibits p65 acetylation-dependent NF-*k*B activation [94] and in the pathogenesis of diabetic retinopathy, activation of NF-*k*B is considered to accelerate apoptosis of capillary cells, suggesting that inhibitor has potential to inhibit the development of diabetic retinopathy. Histone deacetylases inhibitor can suppress the growth and/or survival of tumor cells [95, 96], and Vorinostat and Romidepsin are the first and only histone deacetylases inhibitors approved by FDA for clinical use [97]. However, other inhibitors of histone deacetylases are in clinical trials for other types of cancer treatment. 

 Since miRNAs can regulate gene expression via different ways, including translational repression, mRNA cleavage, and deadenylation, miRNAs have rapidly emerged as promising targets for the development of novel therapeutics. Double-stranded miRNA mimics and antimRNA antisense oligodeoxyribonucleotide are the two commonly investigated techniques to target specific miRNA. miRNAs have a specific and defined target in the pathogenic mechanism of the disease, miRNA-based therapy comes with the advantage that they target multiple genes involved in the same pathway process [98]. One of the major caveats with the miRNA-based therapy is their delivery, as these modulators must leave the circulatory system to get into the target tissue and should be able to cross blood-retina barrier. The other important issue is their circulatory half-life. The advances in drug delivery techniques, however, could open up the use of miRNAs for diabetic retinopathy.

 As mentioned above, epigenetic modifications are influenced by environmental and dietary factors; many natural compounds have been tested to evaluate their beneficial effects on such modifications [99, 100]. Polyphenols, including resveratrol, a natural compound found in the skin of red grapes and a constituent of red wine, are implicated in the regulation of histone deacetylases, Sirt1 [101]. Curcumin (diferuloylmethane), a natural compound commonly used as a curry spice, is shown to modulate a number of histone modifying enzymes and miRNAs [102, 103], and our previous work has shown that its curcumin ameliorates retinal metabolic abnormalities postulated to be important in the development of diabetic retinopathy [104]. Thus, natural compounds could have potential benefits in inhibiting the development of retinopathy in diabetic patients via modulating both metabolic abnormalities and epigenetic modifications.

The role of epigenetic modifications in the pathogenesis of diabetic retinopathy is an emerging area. Recent research has shown that the diabetes environment fosters epigenetic modifications of a number of genes implicated in the pathogenesis of diabetic retinopathy, and enzymes responsible for bringing in the epigenetic changes are altered. The use of the inhibitors and mimics is targeted to regulate the histone modification, DNA methylation and miRNAs are being targeted for a number of chronic diseases [105–107], and the recent advances in the drug delivery to the retina are now opening up the field for their future testing for this devastating disease which a diabetic patient fears the most.
